# Effect of prolonged sitting with stair climbing exercise “snacks” on circulating myokines profiles

**DOI:** 10.1007/s00421-026-06229-2

**Published:** 2026-04-10

**Authors:** Shumpei Fujie, Hossein Rafiei, Michiko Kanemoto, Satoshi Konishi, Motoyuki Iemitsu, Jonathan P. Little

**Affiliations:** 1 Ritsumeikan-Global Innovation Research Organization, Ritsumeikan University, 1-1-1 Nojihigashi, Kusatsu, Shiga 525-8577 Japan; 2 Department of Nutrition and Dietetics, Saint Louis University, Saint Louis, MO USA; 3 Faculty of Sport and Health Science, Ritsumeikan University, Shiga, Japan; 4 Department of Mechanical Engineering, College of Science and Engineering, Ritsumeikan University, Shiga, Japan; 5 School of Health and Exercise Sciences, University of British Columbia, Okanagan Campus, Kelowna, BC Canada

**Keywords:** Stair climbing, Acute exercise, Myokine, Apelin

## Abstract

**Purpose:**

Recent studies have reported positive cardiometabolic responses when prolonged sitting is broken up with brief, bursts of vigorous activity termed “exercise snacks”. However, the mechanism underlying the potential health benefits of exercise snacks remain unstudied. Exercise-induced muscle contractions can promote the release of myokines that contribute to muscle remodeling and systemic cardiometabolic adaptations through inter-organ signaling. This study aimed to profile the effects of stair-climbing exercise snacks on secretion of six established myokines in young normal-weight males and adults living with overweight or obesity.

**Methods:**

In two separate randomized crossover studies, 11 young normal-weight men (NW; study 1) and 8 adults with overweight/obesity (OW; study 2) completed two experimental conditions: (i) sedentary (SED; 9-h sitting) and (ii) exercise snacks (SS; 15–30 s stair-climbing once per hour). The same high-glycemic index meals were consumed at 0, 3, and 6 h at each condition. The primary outcome was the changes in circulating myokines levels before and after the 9-h.

**Results:**

Changes in circulating apelin levels from baseline to 9-h were higher in the SS condition compared to SED in both NW (SS: 102.2 ± 223.7 pg/mL, SED: -59.9 ± 131.6 pg/mL; *P* < 0.05) and OW (SS: 46.4 ± 85.3 pg/mL, SED: -120.3 ± 194.2 pg/mL; *P* < 0.05) groups, with no apparent difference between groups. Plasma concentration of other myokines remained unchanged across 9-h in SED and SS in both groups.

**Conclusions:**

Hourly stair-climbing exercise snacks did not induce clear alterations in most measured myokines, whereas apelin levels were elevated across the day compared with prolonged sitting in both groups.

## Introduction

Physical inactivity is a major global health concern, and 31% of the world’s adult population, or 1.8 billion adults, are classified as physically inactive (Kohl et al. 2012; Strain et al. 2024). In addition to insufficient physical activity, sedentary behavior, defined as low energy expenditure (≤ 1.5 METs) while sitting, reclining, or lying down during waking hours, has become increasingly common in contemporary societies (Ferreira-Santos et al. 2024). Sedentary behavior, irrespective of physical activity levels, has been identified as an independent risk factor for cardiovascular disease and mortality (Biswas et al. 2015). Common barriers to regular exercise include perceived lack of time and limited access to equipment or facilities (Hoare et al. 2017; Trost et al. 2002). Consequently, time-efficient, equipment-free strategies that promote physical activity and interrupt prolonged sedentary behavior offer significant potential to reduce mortality risk and healthcare costs. Therefore, the development of brief and practical exercise interventions that can be easily implemented hold potential for improving health.

The concept of “exercise snacks” involving brief (< 1-min) bursts of vigorous activity, interspersed with 1–4 h of recovery and requiring no specialized equipment or supervision, is increasingly recognized for its potential health benefits (Weston et al. 2025). Previous work has shown that breaking up 9 h of prolonged sitting with hourly ~ 30-s stair climbing exercise snacks lowered postprandial insulin and plasma non-esterified fatty acid levels in adults with overweight or obesity (Rafiei et al. 2021). Additionally, breaking up prolonged sitting with hourly stair climbing exercise snacks improved resting femoral artery shear patterns (Caldwell et al. 2021). Furthermore, a systematic review shows that the repeated bouts of acute exercise snacks contribute to chronic improvements of human health (Weston et al. 2025). Although the physiological benefits of exercise snacks are gradually being elucidated, their underlying mechanisms remain unclear. Exercise-induced skeletal muscle contractions trigger myokine release, which regulates muscle mass, function, and regeneration by modulating protein synthesis, insulin sensitivity, lipid oxidation, myogenesis, mitochondrial biogenesis, autophagy, mitophagy, and extracellular matrix remodeling (Hoffmann et al. 2017; Laurens et al. 2020; Piccirillo et al. 2019; Sabaratnam et al. 2022). Additionally, myokines mediate systemic cardiometabolic adaptations via inter-organ communication, influencing adipose tissue, liver, and heart function to support glucose homeostasis, white adipose tissue browning, and cardioprotection (Hoffmann et al. 2017; Bay et al. 2020; Chow et al. 2022). While many exercise-responsive myokines have been identified (Khan et al. 2019), it remains unclear whether brief and intermittent activity, such as exercise snacks, can promote an increase in circulating myokine levels.

Herein, we hypothesized that the breaking up prolonged sitting with hourly short stair climbing exercise snacks would alter circulating myokines levels compared with a 9-h period of continuous sitting. To test this hypothesis, we performed an exploratory secondary analysis profiling six established myokines in plasma samples collected in a previously published randomized crossover experiment in young normal-weight males and adults with overweight or obesity (Rafiei et al. 2021).

## Methods

### Study design and ethical approval

**Fig. 1:**
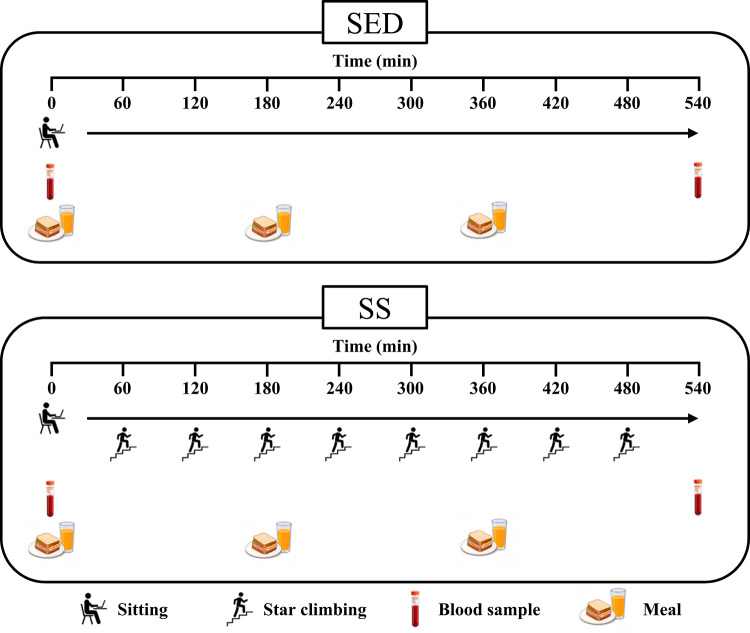
Overview of experimental study design. SED, sedentary; SS, stair snacks; Meal, high-glycemic index meal

A randomized crossover design was used involving two 9-h experimental trials: i) sedentary (SED; participants were sitting on a chair throughout the experimental trial and asked to minimize their movement) and ii) stair climbing exercise snacks (SS; ascending three flights of stairs at a brisk speed (15–30 s) once every hour (× 8)) with identical meals (Fig. 1). Stair climbing was chosen as a model activity because it is a practical, functional form of intermittent physical activity that can be readily implemented in daily life. Details of the clinical trial have been published previously (Rafiei et al. 2021), and this study is conducted as secondary analysis of the research. In SED and SS, participants received the same high–glycemic index meals (peanut butter–jelly sandwich and 400 mL orange juice; ~ 530 kcal: 97 g carbohydrate, 11 g protein, 11 g fat) at 0, 180, and 360 min. A 3–7-day washout separated each trial. The study was approved by the University of British Columbia Clinical Research Ethics Board (ID H17-01747) and was registered on ClinicalTrials.gov (NCT03374436). The study conformed to the standards set by the Declaration of Helsinki. All participants provided written informed consent before data collection.

### Participants

Participants were recruited via posters, e-mail, and word of mouth on the University of British Columbia (Kelowna, Canada) campus. Two groups were studied: young normal-weight men (Study 1; n = 11; age 23.1 ± 4.4 yr; height 175.9 ± 5.7 cm; body weight 74.7 ± 5.9 kg; BMI 24.2 ± 2.1 kg/m^2^; waist circumference 80.2 ± 4.9 cm; resting heart rate 62.0 ± 11.7 bpm) and adults with overweight/obesity (Study 2; n = 8; waist circumference > 88 cm for women or > 102 cm for men; age 52.3 ± 12.4 yr; height 171.3 ± 9.4 cm; body weight 101.6 ± 21.4 kg; BMI 34.5 ± 6.6 kg/m^2^; waist circumference 107.9 ± 10.7 cm; resting heart rate 67.0 ± 11.2 bpm). Inclusion criteria were: (Study 1) age 18–35 yr with BMI 18.5–24.9 kg/m^2^; (Study 2) age 18–69 yr with waist circumference ≥ 88 cm (women) or ≥ 102 cm (men). Exclusion criteria for both were: diagnosed diabetes, use of insulin/oral hypoglycemics or other glucose‐affecting medications, cardiovascular disease, smoking, egg/peanut allergy, serious (> 5 d·wk⁻^1^) exercise training, medical/orthopedic conditions limiting activity, or adherence to special diets (e.g., ketogenic or vegan).

### Experimental trials

Participants met with a dietician pre-trial to confirm no special diets or recent weight changes. They completed a 24-h food recall before the first period of the crossover design and replicated that same intake the day prior to subsequent trials, which the dietician verified each morning. Participants arrived at the laboratory between 7:00 and 8:30 AM after an overnight fast (≥ 10 h). During the first visit, participants provided written informed consent and underwent a lifestyle and medical interview by a registered dietician to confirm eligibility. Anthropometric measurements, including height, weight, waist, and hip circumferences, were taken, with circumferences measured in duplicate to the nearest 0.5 cm following standard protocols. An intravenous catheter was placed in an antecubital vein for repeated blood sampling at fasting (time 0) and over 540 min. Samples were centrifuged at 1550 g for 15 min at 4 °C and stored at − 80 °C until batch analysis. During the 9-h trials, participants remained seated, engaged in sedentary activities, with brief (~ 10 m walk) bathroom breaks allowed. Meals were provided immediately after the first blood draw and at 180 and 360 min, with water ad libitum. In the SS condition, participants walked ~ 25 m to an adjacent stairwell and ascended 55 steps (three flights) as quickly and safely as possible every hour starting at 60 min, totaling eight bouts, with stair climbs at 180 and 360 min performed immediately before meals.

### Myokine measurements

The plasma myokine concentrations were measured using a Luminex multiplex assay kit with magnetic beads (HMYOMAG-56 K MILLIPLEX MAP Human Myokine Magnetic Bead Panel, MilliporeSigma, Oakville, ON, Canada) (Seok et al. 2023). The beads were conjugated with the antibodies for the following 6 myokines identified in previous studies: apelin, brain-derived neurotrophic factor (BDNF), fibroblast growth factor 21 (FGF 21), follistatin-like 1 (FSTL-1), musclin, and secreted protein acidic rich in cysteine (SPARC). Briefly, the beads were incubated using diluted plasma from young normal-weight males and adults with overweight or obesity, washed with a buffer, and measured on a Luminex 100/200 instrument (Luminex, Austin, TX, USA). The results of the Luminex multiplex assay were obtained as median fluorescence intensity (MFI) values. The coefficients of variation for duplicate measurements were 6.1 ± 4.7% for apelin, 12.4 ± 7.3% for BDNF, 11.0 ± 11.0% for FGF21, 9.7 ± 8.8% for FSTL-1, 6.2 ± 6.7% for musclin and 4.9 ± 3.8% for SPARC.

### Statistical analysis

Values are expressed as mean ± standard deviation (SD). Given the studies were conducted at separate times and the groups were not matched for sex or age, a two-way repeated-measures ANOVA was performed separately within each group to assess condition and time effects. In addition, separate unpaired t-tests were used to assess changes in each parameter by calculating the change (delta [Δ] from time 540 min minus time 0) in the SED and SS conditions, with analyses conducted separately within each group. To control for multiple comparisons, p-values are presented both uncorrected and after adjustment using the Holm–Bonferroni method. Statistical significance was set at *P* < 0.05. All statistical analyses were performed using StatView (version 5.0, SAS Institute, Tokyo, Japan), after confirming that all data were normally distributed using the Kolmogorov–Smirnov test. Cohen’s *dz* was calculated by dividing the mean of the paired differences by the standard deviation of those differences. Effect sizes (*dz*) were interpreted according to Cohen’s conventional thresholds (small = 0.2, medium = 0.5, large = 0.8), applied as general guidelines (Cohen et al. 1988). Post hoc statistical power was calculated using G*Power (version 3.1.9.7; Franz Faul, Universität Kiel, Germany) based on the observed effect size (Cohen’s *dz*), α = 0.05, and the actual sample size.

## Results

### Changes in circulating myokine levels by snacks in normal-weight males (Study 1)

**Fig. 2:**
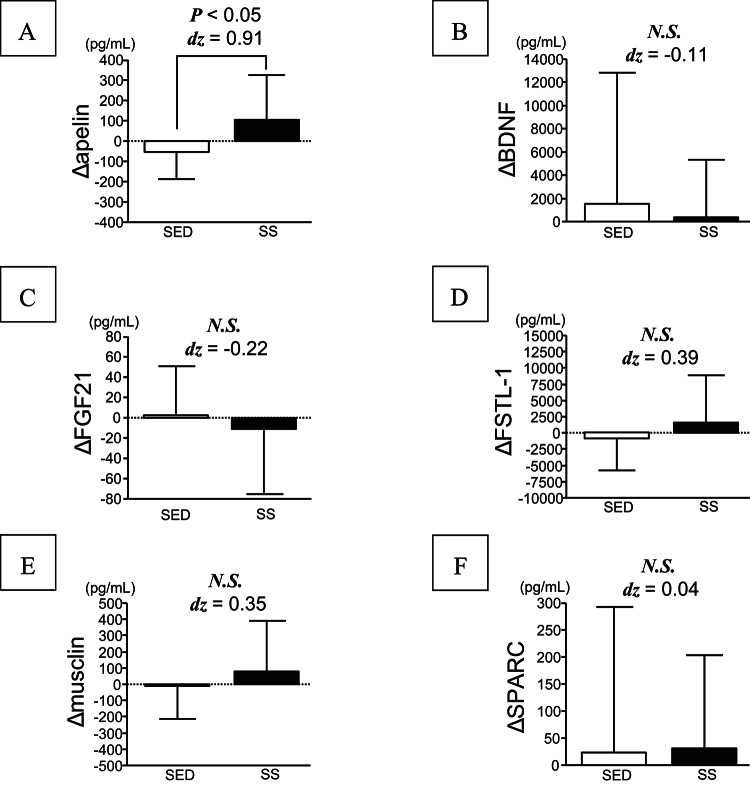
Comparison of changes in plasma apelin (**A**), BDNF (**B**), FGF21 (**C**), FSTL-1 (**D**), musclin (**E**), and SPARC (**F**) levels before and after the SED or SS conditions in the normal-weight males. Δ: changes in value before and after interventions. Data are expressed as mean ± SD. BDNF, brain-derived neurotrophic factor; FGF, fibroblast growth factor; FSTL, follistatin-like; SPARC, secreted protein acidic rich in cysteine; SED, sedentary; SS, stair snacks; SD, standard deviation

No significant differences between groups or times were noted for circulating apelin, BDNF, FGF21, FSTL1, musclin and SPARC levels in normal-weight males (Table 1). Changes in circulating apelin levels across the 9-h study visit were higher in the SS condition when compared to the SED condition in normal-weight males (*P* < 0.05, *dz* = 0.91, power = 0.78, Fig. 2A), although this difference did not remain significant after Holm-Bonferroni correction. However, changes in circulating BDNF, FGF21, FSTL-1, musclin, and SPARC levels were not significant between the two conditions (Fig. 2B–F).

**Table 1:** Comparison of circulating myokines before and after the intervention in normal-weight males

	SED		SS		*P* value (Groups x Time)
	Pre	Post	Pre	Post	
Apelin, pg/mL	322.4 ± 116.2	262.4 ± 130.0	347.0 ± 111.1	449.3 ± 197.2	0.0668
BDNF, pg/mL	6440.0 ± 5134.5	7936.2 ± 13168.7	5331.5 ± 5650.0	5630.7 ± 5257.4	0.8065
FGF21, pg/mL	56.2 ± 22.5	58.9 ± 50.5	83.1 ± 67.5	56.2 ± 22.5	0.5968
FSTL1, pg/mL	13185.0 ± 5761.8	12133.3 ± 6542.9	14596.5 ± 5663.9	16005.6 ± 7552.2	0.5289
Musclin, pg/mL	937.8 ± 465.7	921.2 ± 542.4	992.4 ± 392.1	1072.0 ± 514.2	0.7422
SPARC, pg/mL	423.9 ± 244.7	447.3 ± 312.6	404.5 ± 220.1	435.7 ± 69.5	0.9592

Values are means and SD

*SED* sedentary, *SS* stair snacks, *BDNF* brain-derived neurotrophic factor, *FGF21* fibroblast growth factor-21, *FSTL1*: follistatin-like 1, *SPARC* secreted protein acidic and rich in cysteine

### Changes in circulating myokine levels by snacks in adults with overweight/obesity (Study 2)

**Fig. 3:**
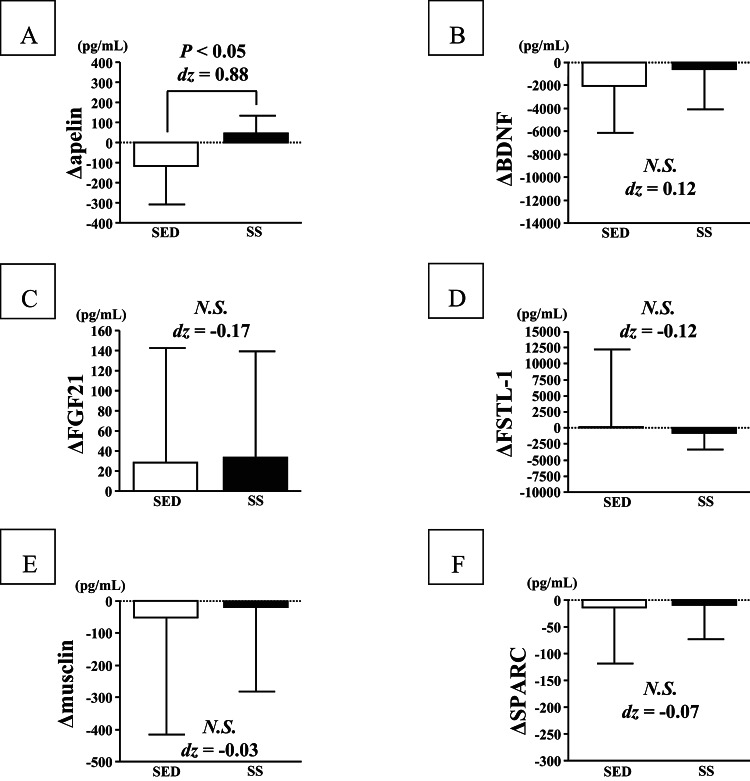
Comparison of changes in plasma apelin (**A**), BDNF (**B**), FGF21 (**C**), FSTL-1 (**D**), musclin (**E**), and SPARC (**F**) levels before and after the SED or SS conditions in the adults with overweight/obesity. Δ: changes in value before and after interventions. Data are expressed as mean ± SD. BDNF, brain-derived neurotrophic factor; FGF, fibroblast growth factor; FSTL, follistatin-like; SPARC, secreted protein acidic rich in cysteine; SED, sedentary; SS, stair snacks; SD, standard deviation

No significant differences between groups or times were noted for circulating apelin, BDNF, FGF21, FSTL1, musclin and SPARC levels in adults with overweight/obesity (Table 2). Similar to Study 1, circulating apelin levels in adults with overweight/obesity increased significantly in the SS condition compared to the SED condition across 9-h (*P* < 0.05, *dz* = 0.88, power = 0.57, Fig. 3A), but these changes did not remain significant after Holm-Bonferroni correction. However, the levels of other myokines, including BDNF, FGF21, FSTL-1, musclin, and SPARC, remained unchanged between the two conditions (Fig. 3B-F).

**Table 2:** Comparison of circulating myokines before and after the intervention in adults with overweight/obesity

	SED		SS		*P* value (Groups x Time)
	Pre	Post	Pre	Post	
Apelin, pg/mL	437.7 ± 182.1	317.4 ± 223.9	332.6 ± 87.2	378.9 ± 111.7	0.1590
BDNF, pg/mL	9020.6 ± 5810.8	6901.7 ± 8085.0	8404.4 ± 4598.9	7729.3 ± 5903.4	0.7514
FGF21, pg/mL	143.7 ± 90.7	171.7 ± 176.8	165.1 ± 109.9	198.5 ± 146.3	0.9569
FSTL1, pg/mL	16234.3 ± 6348.4	16264.6 ± 14121.5	15695.1 ± 3413.7	14805.3 ± 4275.6	0.8757
Musclin, pg/mL	1023.0 ± 301.9	968.9 ± 367.0	956.9 ± 184.8	936.8 ± 206.2	0.8645
SPARC, pg/mL	452.7 ± 307.4	437.6 ± 335.7	436.3 ± 267.6	425.0 ± 233.0	0.9853

Values are means and SD

*SED* sedentary, *SS *stair snacks, *BDNF* brain-derived neurotrophic factor, *FGF21* fibroblast growth factor-21, *FSTL1* follistatin-like 1, *SPARC* secreted protein acidic and rich in cysteine

## Discussion

In this exploratory analysis of six circulating myokines, high-intensity stair-climbing exercise snacks did not significantly alter most measured myokines (BDNF, FGF21, FSTL-1, musclin, and SPARC), with only one (apelin) showing a detectable response. This study reveals that brief, high-intensity stair-climbing exercise snacks may influence plasma apelin levels in both young normal-weight males and adults with overweight or obesity. These results suggest that even minimal doses of strategically applied exercise can modulate circulating apelin concentration, which is a myokine linked to favorable metabolic and cardiovascular outcomes. This evidence highlights the potential for integrating short and practical exercise interventions into sedentary lifestyles and suggests that altered myokines could be involved in the acute metabolic response to exercise snacks in humans.

In this study, circulating apelin levels were higher in both young normal-weight males and adults with overweight or obesity following hourly stair climbing exercise snacks compared to prolonged sitting condition. Previous research has shown that acute high-intensity sprint interval training induces a transient elevation in circulating apelin levels (Kon et al. 2021), while chronic exercise leads to a sustained increase in basal apelin levels (Fujie et al. 2014). These findings suggest that the periodic increases in apelin levels triggered by acute exercise may cumulatively contribute to the enhanced basal apelin levels observed with chronic exercise. Previous studies have identified apelin as a promising target for developing novel therapeutic strategies for hypertension and cardiovascular diseases (Yamaleyeva et al. 2016). Additionally, apelin has been shown to regulate glucose and lipid metabolism in target tissues such as skeletal muscle and adipose tissue (Bertrand et al. 2015). Furthermore, apelin has been recognized as both a diagnostic marker for early sarcopenia and a potential pharmacological target to prevent age-related muscle weakness and restore physical autonomy (Vinel et al. 2018). Therefore, the exercise snacks-induced elevation of apelin levels may contribute to improving metabolic and cardiovascular health.

Several myokines in plasma were not changed after exercise snacks in this study. The secretion of myokines differs between exercise types, exercise intensities, duration and other factors. A previous meta-analysis indicates that small to large increases can be induced by a single bout of exercise, especially when blood is collected immediately after and up to 60 min post-exercise (Bettariga et al. 2024). Additionally, both aerobic and resistance exercise induce alterations in myokine expression, although the extent of these changes varies depending on the specific myokine (Bettariga et al. 2024). The positive effect of endurance exercise on BDNF levels appears to be intensity dependent (Ferris et al. 2007), and meta-analysis shows that high-intensity exercise significantly increases circulating BDNF levels compared to light-intensity exercise (Fernández-Rodríguez et al. 2022). Additionally, acute high-intensity interval exercise (HIIE) effectively increases circulating BDNF, FGF21, and FSTL-1 levels compared to moderate-intensity continuous exercise (MICE), suggesting that the increases in these myokines are dependent on exercise intensity (Ji et al. 2024). In that study, the responses of these factors followed an intensity-dependent order (moderate, vigorous, and high) and were positively correlated with changes in blood lactate concentrations before and after exercise, indicating that their elevations are indeed intensity-dependent (Ji et al. 2024). In addition, exercise at an intensity of 60% peak oxygen uptake (VO_2peak_) significantly increases circulating SPARC levels, whereas exercise of 40% VO_2peak_ does not (Miyamoto et al. 2022). The exercise snacks in this study therefore may have lacked sufficient exercise intensity and/or duration to induce changes in BDNF, SPARC, FSTL-1, FGF 21, and musclin levels in plasma. On the other hand, the apelin concentration may have increased after a single bout of exercise, and this stimulation, when performed regularly at 60-min intervals, could have accumulated, leading to a sustained elevation even after 540 min.

In this study, circulating apelin levels increased after exercise snacks in both normal-weight males and adults with overweight or obesity. Additionally, there were no significant differences in the changes in apelin levels between normal-weight males and adults with overweight or obesity. These findings indicate that exercise snacks appear similarly efficacious for increasing apelin levels across individuals with different body compositions. Conversely, previous studies have demonstrated that circulating apelin concentrations vary among clinical populations. A meta-analysis in previous study reported that circulating apelin levels were increased in people living with type 2 diabetes with impaired glycemic control (Noori-Zadeh et al. 2019). Another meta-analysis showed that lower circulating apelin levels were significantly associated with an increased risk of hypertension (Xie et al. 2017). Therefore, future studies are needed to examine whether exercise snacks can increase circulating apelin levels in adults with cardiometabolic disease (e.g., type 2 diabetes and hypertension) as seen in this study.

The strengths of the present study include its randomized crossover design and the implementation of a controlled yet ecologically valid intervention incorporating prolonged sitting and stair climbing, which reflects real-world behavioral patterns. This design minimized inter-individual variability and enabled a rigorous evaluation of acute physiological responses under practical conditions. However, several limitations should be acknowledged; therefore, these results should be interpreted with caution. First, the current analyses were secondary in nature, and the study was not specifically powered to detect changes in circulating myokines. Additionally, although the effect sizes of apelin were large in both groups, the relatively modest sample size may have reduced statistical power and thus limited the interpretability of the findings. In fact, after Holm-Bonferroni correction, the changes in apelin levels did not remain significant. Second, although alterations in plasma myokine (apelin) concentrations were observed, we were unable to directly confirm their release from skeletal muscle. Additionally, the blood sampling was limited to baseline and 540 min, precluding detailed assessment of the temporal kinetics of individual myokines. Because the half-lives of the measured myokines vary, differences in sampling timing may have affected the observed responses. Finally, the study design did not allow for an examination of potential sex differences, which should be addressed in future research.

## Conclusions

Of the six myokines measured, five (BDNF, FGF21, FSTL-1, musclin, and SPARC) showed no detectable change in response to hourly stair climbing exercise snacks, whereas only one (apelin) showed a potential response. Hourly stair climbing exercise snacks may lead to an increase plasma apelin levels in normal-weight males and adults with overweight or obesity when compared to 9 h of prolonged sitting. These findings suggest that exercise snacks may alter select myokines in humans, potentially helping to uncover mechanisms underlying the cardiometabolic effects of short bursts of vigorous exercise.

## Data Availability

The data that supports the findings of this study are available from the corresponding author upon reasonable request.

## Abbreviations

SED: Sedentary
SS: Stair climbing exercise snacks
BMI: Body mass index
BDNF: Brain-derived neurotrophic factor
FGF21: Fibroblast growth factor 21
FSTL-1: Follistatin-like 1
SPARC: Secreted protein acidic rich in cysteine
MFI: Median fluorescence intensity
SD: Standard deviation
HIIE: High-intensity interval exercise
MICE: Moderate-intensity continuous exercise
VO_2peak_: Peak oxygen uptake
